# The Interrelationship between Sarcoidosis and Atherosclerosis—Complex Yet Rational

**DOI:** 10.3390/jcm11020433

**Published:** 2022-01-15

**Authors:** Sara Hoss, Tzlil Grinberg, Alon Eisen

**Affiliations:** 1 Department of Cardiology, Rabin Medical Center, Petah Tikva 4941492, Israel; tzlilgrin@gmail.com (T.G.); alon201273@gmail.com (A.E.); 2 Sackler Faculty of Medicine, Tel Aviv University, Tel Aviv 6139001, Israel

Sarcoidosis is a systemic inflammatory disease of unknown etiology, characterized by the presence of non-caseating granulomas in affected organs. Its clinical course is variable and ranges between mild asymptomatic disease and multi-organ involvement causing significant morbidity and mortality [1]. Cardiac sarcoidosis is clinically diagnosed in approximately 5% of patients with systemic sarcoidosis. However, multiple studies suggest that its true incidence is higher, as histopathology from autopsies reveals myocardial involvement in over 25% of patients [2,3,4]. The clinical manifestations vary, ranging from conduction abnormalities and tachyarrhythmia to heart failure and sudden death, depending on the extent and location of myocardial inflammation [5]. Although atherosclerosis is not a typical manifestation of cardiac sarcoidosis, recent data unveil a possible association between the two.

Over the years, the role of inflammatory mechanisms in atherosclerosis has been established. A cellular immune response plays a central role in the atherosclerotic process, as inflammatory cells including macrophages and lymphocytes infiltrate the vascular tissue and release proinflammatory cytokines, such as interleukins (ILs), tumor necrosis factor α (TNFα), and platelet-derived growth factor (PDGF). The cytokines compromise endothelial function and mediate the migration of inflammatory cells into the plaque. Inflammatory cytokines also play a role in advanced stages of the process, such as plaque rupture and atherothrombosis [6,7].

In recent years, chronic inflammation has been recognized as a new, non-traditional, risk factor for atherosclerosis. Studies on various chronic inflammatory diseases have demonstrated increased morbidity and mortality due to cardiovascular disease (CVD) often related to accelerated atherosclerosis. In patients with systemic lupus erythematosus (SLE), for example, the overall risk of myocardial infarction (MI) is 10-fold higher than that in the general population, even after accounting for traditional Framingham risk factors. Higher cardiovascular morbidity and mortality not explained by traditional risk factors were also evident in rheumatoid arthritis, systemic sclerosis, and Sjögren’s syndrome [8,9,10]. The mechanisms behind the accelerated atherosclerosis in systemic inflammatory diseases are not completely understood, but the release of inflammatory cytokines in systemic inflammation as well as high levels of various inflammatory cells and autoantibodies have been suggested as possible mechanisms.

To date, several studies have investigated the association between sarcoidosis and atherosclerosis. A study by Yilmaz et al., on 74 sarcoidosis patients, demonstrated increased carotid intima–media thickness (CIMT) and decreased flow-mediated dilatation (FMD) compared with healthy volunteers, indicating the presence of endothelial dysfunction and subclinical atherosclerosis [11]. Another study supported that hypothesis by demonstrating 40 sarcoidosis patients who had lower coronary flow velocity reserve (CFVR) compared to controls [12]. Population-based retrospective studies demonstrated conflicting data; a recent meta-analysis found no clear association between sarcoidosis and ischemic heart disease (IHD), as opposed to a study by Ungprasert et al., which demonstrated an increased risk of CVD in sarcoidosis patients compared with matched comparators [13,14]. The latter conclusion is reinforced by the present publication by Gonen et al., which demonstrates a robust association between sarcoidosis and IHD in a large cohort of 3750 sarcoidosis patients. Another important finding is the increased all-cause mortality of patients with coexisting sarcoidosis and IHD compared with patients with only one of these conditions, as well as healthy matched controls. Both conclusions emphasize the need for the active screening and aggressive management of traditional risk factors in patients with sarcoidosis.

Despite its retrospective design and obvious limitations, the study by Gonen et al., together with prior studies, may have important clinical implications. Recent studies suggest that targeted therapy for specific inflammatory diseases may be associated with a reduced risk of CVD, for example, hydroxychloroquine therapy for SLE patients [15]. Although a similar effect in sarcoidosis has not been described to date, other anti-inflammatory treatments, such as colchicine, may be of interest and deserve further research [16]. Finally, despite increasing evidence linking inflammatory diseases, including sarcoidosis, to CVD, data regarding the nature of this association and specific mechanisms involved in it are lacking. A better understanding of these mechanisms could assist in the diagnosis, prevention and treatment of early atherosclerosis and related morbidity in patients with sarcoidosis.
